# Automated 3D segmentation of rotator cuff muscle and fat from longitudinal CT for shoulder arthroplasty evaluation

**DOI:** 10.1007/s00256-025-04991-6

**Published:** 2025-08-09

**Authors:** Mingrui Yang, Bong-Jae Jun, Tammy Owings, Nikhil Subhas, Joshua Polster, Carl S. Winalski, Jason C. Ho, Vahid Entezari, Kathleen A. Derwin, Eric T. Ricchetti, Xiaojuan Li

**Affiliations:** 1 Department of Biomedical Engineering, Lerner Research Institute, Cleveland Clinic, 9500 Euclid Avenue, Cleveland, OH 44195 USA; 2 Department of Radiology, Imaging Institute, Cleveland Clinic, Cleveland, OH USA; 3 Department of Orthopaedics, Cleveland Clinic, Cleveland, OH USA; 4 Program of Advanced Musculoskeletal Imaging (PAMI), Cleveland Clinic, Cleveland, OH USA

**Keywords:** Deep learning, Automated segmentation, Rotator cuff muscle, Muscle volume, Fat fraction, Computed tomography, Total shoulder arthroplasty

## Abstract

**Objective:**

To develop and validate a deep learning model for automated 3D segmentation of rotator cuff muscles on longitudinal CT scans to quantify muscle volume and fat fraction in patients undergoing total shoulder arthroplasty (TSA).

**Methods:**

The proposed segmentation models adopted DeepLabV3 + with ResNet50 as the backbone. The models were trained, validated, and tested on preoperative or minimum 2-year follow-up CT scans from 53 TSA subjects. 3D Dice similarity scores, average symmetric surface distance (ASSD), 95th percentile Hausdorff distance (HD95), and relative absolute volume difference (RAVD) were used to evaluate the model performance on hold-out test sets. The trained models were applied to a cohort of 172 patients to quantify rotator cuff muscle volumes and fat fractions across preoperative and minimum 2- and 5-year follow-ups.

**Results:**

Compared to the ground truth, the models achieved mean Dice of 0.928 and 0.916, mean ASSD of 0.844 mm and 1.028 mm, mean HD95 of 3.071 mm and 4.173 mm, and mean RAVD of 0.025 and 0.068 on the hold-out test sets for the pre-operative and the minimum 2-year follow-up CT scans, respectively.

**Conclusion:**

This study developed accurate and reliable deep learning models for automated 3D segmentation of rotator cuff muscles on clinical CT scans in TSA patients. These models substantially reduce the time required for muscle volume and fat fraction analysis and provide a practical tool for investigating how rotator cuff muscle health relates to surgical outcomes. This has the potential to inform patient selection, rehabilitation planning, and surgical decision-making in TSA and RCR.

## Introduction

Shoulder disorders such as rotator cuff tear and osteoarthritis are among the most significant musculoskeletal disorders that cause pain and impair daily functionality [1]. There has been a significant increase in the number of surgical procedures performed for shoulder disorders and their associated annual cost to the healthcare system in the USA during the past decade [2, 3]. For instance, at least 250,000 rotator cuff repair (RCR) [4] and over 100,000 total shoulder arthroplasty (TSA) surgeries [5] are performed annually in the USA, at an annual healthcare cost burden of up to $3.4 billion dollars [6, 7]. The clinical outcomes, however, are mixed. For instance, patient-reported outcome measures (PROMs) show reliable and substantial short-term improvements in pre-operative pain and shoulder function after RCR. However, at least 20–30% of RCRs experience structural failures [8]. Similarly, while PROMs show reliable and substantial improvement following TSA, outcomes can vary by various factors [9–21], including rotator cuff status [19, 22].

Clinical and basic science studies have shown increasing evidence that muscle volume and quality play significant roles in the prognosis and treatment of shoulder disorders [23–25]. For instance, muscle atrophy and fatty infiltration have been shown to be significantly correlated with worse clinical outcomes after RCR and TSA [23, 24]. Since muscle pathology is potentially modifiable (with exercise training or pharmacological interventions) and can impact surgical decision-making, including the type of surgical repair or shoulder arthroplasty chosen, reliable methods to quantify muscle volume and fat fraction will provide physicians powerful tools to optimize patient management and improve patient outcomes in RCR and TSA [25]. These quantitative measures, however, heavily depend on accurate muscle segmentation, which is extremely challenging and laborious and subject to intra- and inter-reader variation when performed manually [26].

With the rapid evolution of deep learning models in the past couple of decades, researchers have developed various deep learning algorithms to try to automate the rotator cuff muscle segmentation process on magnetic resonance images (MRI) with impressive performance [27–30]. However, apart from the high cost, the current clinical MRI sequences typically lack coverage of the entire rotator cuff muscle volumes of the rotator cuff and have a large slice thickness. Accurate muscle volume and fat fraction quantification on MRI would require high-resolution scans with smaller slice thickness and more advanced MRI sequences such as 6-point Dixon [31, 32], which have not been incorporated in standard clinical routines. Compared to MRI, computed tomography (CT) is less expensive and provides high-resolution images with full coverage of the entire shoulder girdle muscle volume. In addition, through its inherent attenuation values measured in Hounsfield units (HU), CT provides a practical and effective method for estimating muscle fatty infiltration, offering valuable quantitative insights into the evolution of pathologic muscle composition. U-Net has been used to segment rotator cuff muscle groups on a cross-sectional CT slice for fatty infiltration evaluation [26]. However, existing methods have been limited in providing a comprehensive algorithm for accurate quantification of muscle volume and fat fraction on CT images. Our approach sought to address these limitations by achieving high accuracy in these metrics on muscle volume and fat fraction, demonstrating improvements over prior work.

This study aims to build an automated and accurate rotator cuff muscle segmentation model from clinical CT images with deep learning, utilizing a TSA patient cohort to quantify 3D rotator cuff muscle volumes and fat fraction both pre- and postoperatively. This would be the first step towards building reliable automated algorithms using CT imaging to better evaluate the role muscle volume and fat fraction play in predicting clinical outcomes after shoulder procedures like RCR and TSA.

## Methods

### Data

**Fig. 1:**
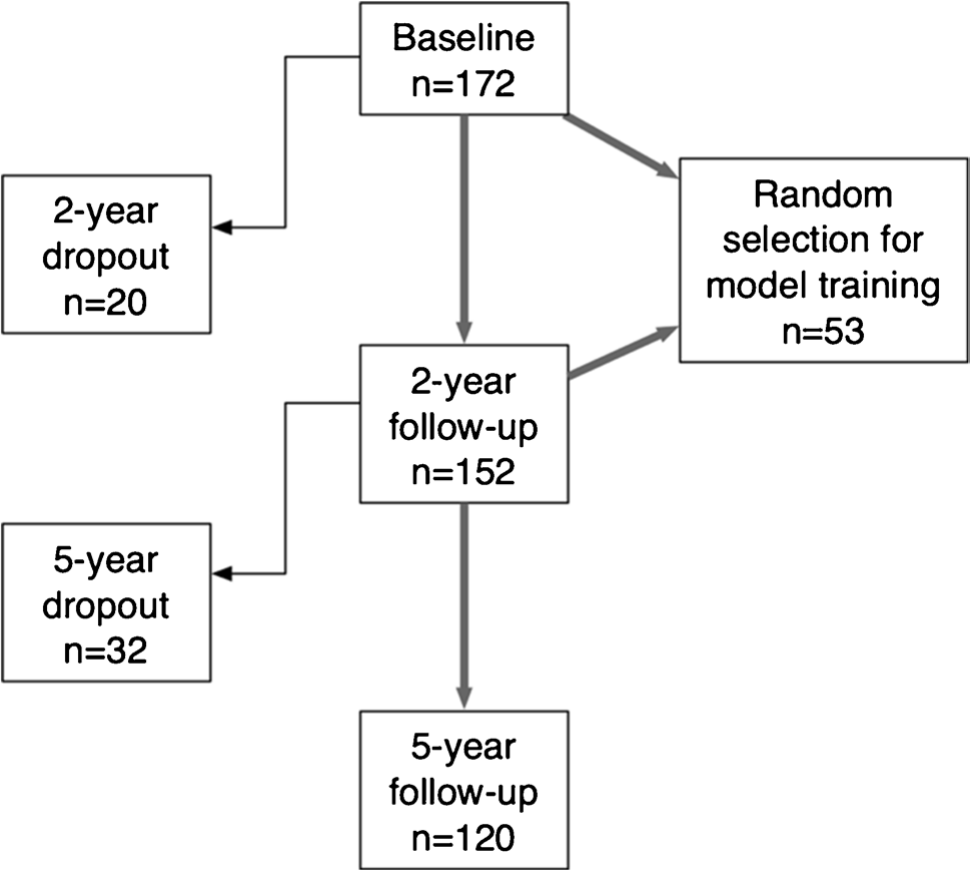
Patient flow chart

This study utilized CT scans from a cohort of 172 patients (55 female with age 63.5 ± 7.6) who underwent TSA and were scanned with sequential CT scans of the operative shoulder at preoperative (T0), minimum 2-year follow-up (T1), and minimum 5-year follow-up (T2) time points as a part of a prospective study protocol evaluating implant positioning following TSA, as previously described [33–36]. All 172 patients underwent preoperative CT imaging, 152 patients underwent CT imaging at minimum 2-year follow-up, and 121 patients underwent CT imaging at minimum 5-year follow-up. A patient flowchart can be found in Fig. 1.

All scans were collected on CT scanners (SOMANTOM Sensation 64, Definition DS, Definition AS +, Definition EDGE, or Definition FLASH; Siemens Healthcare, Erlangen, Germany) using a single-energy protocol with 140 kV (peak), 300 quality reference mA with CARE Dose4D (tube current modulation; Siemens Healthcare), and 0.6-mm collimation. Scans were initially reconstructed using a standard filtered back-projection algorithm in the axial plane with a medium-smooth kernel (B40) and 0.6-mm slice thickness, and postoperative CT scans were further reconstructed using an iterative metal artifact reduction algorithm (iMAR; Siemans, Munich, Germany).

Preoperative and 2-year follow-up CT images from 53 randomly selected TSA patients (14 females; 47 to 85 years old) were used to train two deep learning models for automated rotator cuff muscle segmentation on pre- and postoperative CT images. Once tested and validated, the automated segmentation models were applied to the entire TSA cohort for muscle volume and fat fraction quantification. During this process, CT scan quality was evaluated and 10 preoperative CT scans with excessive noise and beam hardening artifacts were excluded, resulting in 162 preoperative, 152 2-year follow-up, and 121 5-year follow-up CT scans utilized for quantification.

The acquisition and use of these CT scans for research had previously been approved by our Institutional Review Board and obtained under patient informed consent.

### Muscle segmentation

#### Image preprocessing

All scans were reoriented according to the scapular coordinate system defined by three anatomic landmarks (glenoid center, scapular trigonum, inferior angle of the scapular) as previously described [33–36]. The starting and ending slices for muscle volume quantification were the face of the glenoid laterally and the superior angle of the scapula medially. Three rotator cuff muscle groups (supraspinatus, combined infraspinatus/teres minor, and subscapularis) were manually segmented on all slices over the 3D volume by a trained reader (TMO), and the segmentation was reviewed by three experienced shoulder surgeons (ETR, VE, JCH) for consensus agreement. In addition, the trained reader independently segmented the test cases at least 7 months apart from the original. The cases were randomly split into 32/11/10 for DL model training, validation, and testing respectively. CT image intensities were clipped to a range of − 1024 to 1500 Hounsfield units to exclude extreme values and standardize contrast. The resulting values were then shifted to start at zero and saved in 16-bit unsigned integer (uint16) format for deep learning model training, validation, and testing. All CT slices were resized to 512 × 512 pixels. Pixel intensities were then normalized to the range (− 1, 1) by dividing by the intensity range (i.e., 2524 HU after clipping) and shifting the values by subtracting 1.

#### Model architecture

**Fig. 2:**
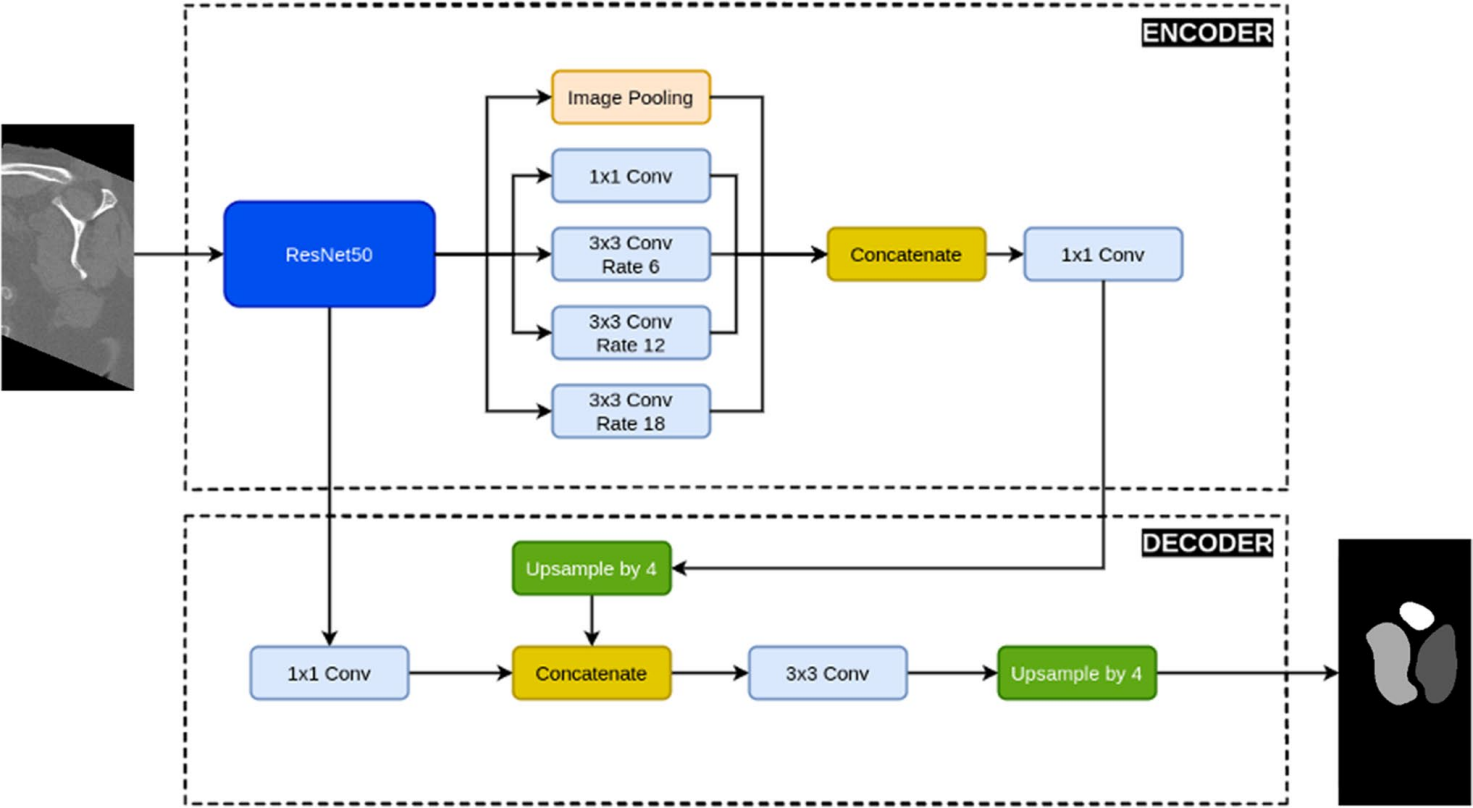
The architecture of the deep learning rotator cuff muscle segmentation model for CT images based on DeepLabV3 + and ResNet50

The deep learning model architecture was based on the DeepLabV3 + [3] with ResNet50 as the backbone. It consisted of an encoder and a decoder. In the encoder, the backbone ResNet50 was fed with the input image. Atrous spatial pyramid pooling (ASPP) with dilation rates of 1, 6, 12, and 18 was then applied to the output features from the fourth stage of ResNet50. These pooled features, along with image-level features obtained from global average pooling, were concatenated and passed through a 1 × 1 convolution kernel to reduce dimensionality. For the decoder, the low-level features from the second convolution layer of the third block in the second stage of ResNet50 were concatenated with the high-level features from the encoder. This was followed by convolutional refinement using 3 × 3 convolutions. A final upsampling step was applied to generate a segmentation mask that matched the input image size. See Fig. 2 for a visualization of the model architecture.

#### Model training, validation, and testing

The Dice coefficient (DC) [37], the average symmetric surface distance (ASSD) [38], the 95th percentile Hausdorff distance (HD95) [39–41], and the relative absolute volume difference (RAVD), all calculated in 3D, were utilized as metrics for model evaluation during training, validation, and testing. These metrics provide a comprehensive assessment of the segmentation quality in terms of both spatial overlap and boundary accuracy.

Specifically, for given ground truth segmentation  $G$ and the target segmentation $S$ to be evaluated, the Dice coefficient is defined as.

$$DC=\frac{2\left|G\cap S\right|}{\left|G\right|+\left|S\right|}$$

The DC is a widely accepted metric for knee tissue segmentation. It ranges from 0 to 1, with 0 indicating no overlap in segmentation and 1 indicating a perfect agreement in segmentation. Volumetric DC was calculated to accurately reflect model segmentation performance.

The Hausdorff distance (HD) [42] between $G$ and $S$ is defined by

$$HD\left(G,S\right)=\text{max}\left(h\left(G,S\right),h\left(S,G\right)\right) ,$$

where the directed Hausdorff distance $h(G,S)$ is given by

$$h\left(G,S\right)=\underset{g\in G}{\text{max}}\underset{s\in S}{\text{min}}\| g-s\| .$$

The HD is an informative metric since it is an indicator of the largest segmentation error. The HD is, however, known to be sensitive to outliers. The HD95 replaces the maximum in HD with the 95 percentiles to reduce the impact of outliers on HD.

The average symmetric surface distance (ASSD) is the average of all distances from points on the boundary of $S$ ($\partial S$) to the boundary of $G$ ($\partial G$) and from points on $\partial G$ to $\partial S$:

$$ASSD\left(G,S\right)=\frac{{\sum }_{g\in \partial G}\underset{s\in \partial S}{\text{min}}\Vert s-g\Vert +{\sum }_{s\in S}\underset{g\in G}{\mathit{min}}\Vert g-s\Vert }{\left|\partial G\right|+\left|\partial S\right|} ,$$

where $\Vert \cdot \Vert$ denotes the Euclidean norm and $|\cdot |$ denotes the cardinality of a set. The smaller the ASSD, the better the segmentation boundaries agree to each other.

The relative absolute volume difference (RAVD) computes the volume difference between $S$ and $G$ relative to the volume of $G$:

$$RAVD= \frac{|S|-|G|}{|G|}$$

Where $|\cdot |$ denotes the cardinality of a set. Note that RAVD has a range of $(-1, +\infty )$, with 0 indicating an ideal score.

In addition to reporting mean performance values of these metrics, we conducted paired *t*-tests to statistically evaluate differences between methods.

Image slices with all three muscle groups segmented were used for model training and validation, resulting in 3587 slices for training and 1303 slices for validation. The dataset was further augmented with Gaussian noise and horizontal flipping to increase variability and robustness, resulting in 14,348 slices for training and 5212 slices for validation. Categorical cross-entropy was employed as the loss function, which is suitable for multi-class segmentation tasks. The model was trained for 100 epochs using the ADAM optimizer with a learning rate of 5e − 5 and a batch size of 16. The weights with the best validation loss on the 11 validation cases were saved and used for model evaluation on the test set.

The performance of the model was evaluated on the ten hold-out test cases and compared against the well-known benchmark 2D U-Net architecture. Additionally, its performance was also benchmarked against intra-reader variation, wherein the trained reader independently segmented the test cases at least 7 months apart from the original segmentation to assess consistency and reliability.

The entire training, validation, and testing processes were implemented using the Python deep learning framework TensorFlow with the Keras API. The computations were performed on an NVIDIA V100s GPU with 32 GB of memory, ensuring efficient processing of large batches and complex models.

### Fat fraction quantification

The trained pre-operative and postoperative DL models were applied to the entire cohort for all three pre- and postop time points for automated segmentation of rotator cuff muscle volume areas on CT images. Based on the literature [43–45], voxels ranging from − 190 to − 30 HU were considered fat and from − 29 HU to + 150 HU as muscle. Therefore, a threshold of − 29 HU was applied to the segmented muscle areas. Areas with HU larger than − 29 were classified as muscle, and those with HU less than or equal to −29 were classified as fat. Note that Hounsfield unit thresholds for fat detection can vary slightly depending on scanner calibration, imaging protocol, and anatomical location. Segmented muscle and fat volumes, as well as fat fraction (FF), were then computed and visualized via violin plots, where.

$$FF= \frac{\text{fat volume}}{\text{fat volume }+\text{ muscle volume}} \times 100\%$$

## Results

### Muscle segmentation

**Fig. 3:**
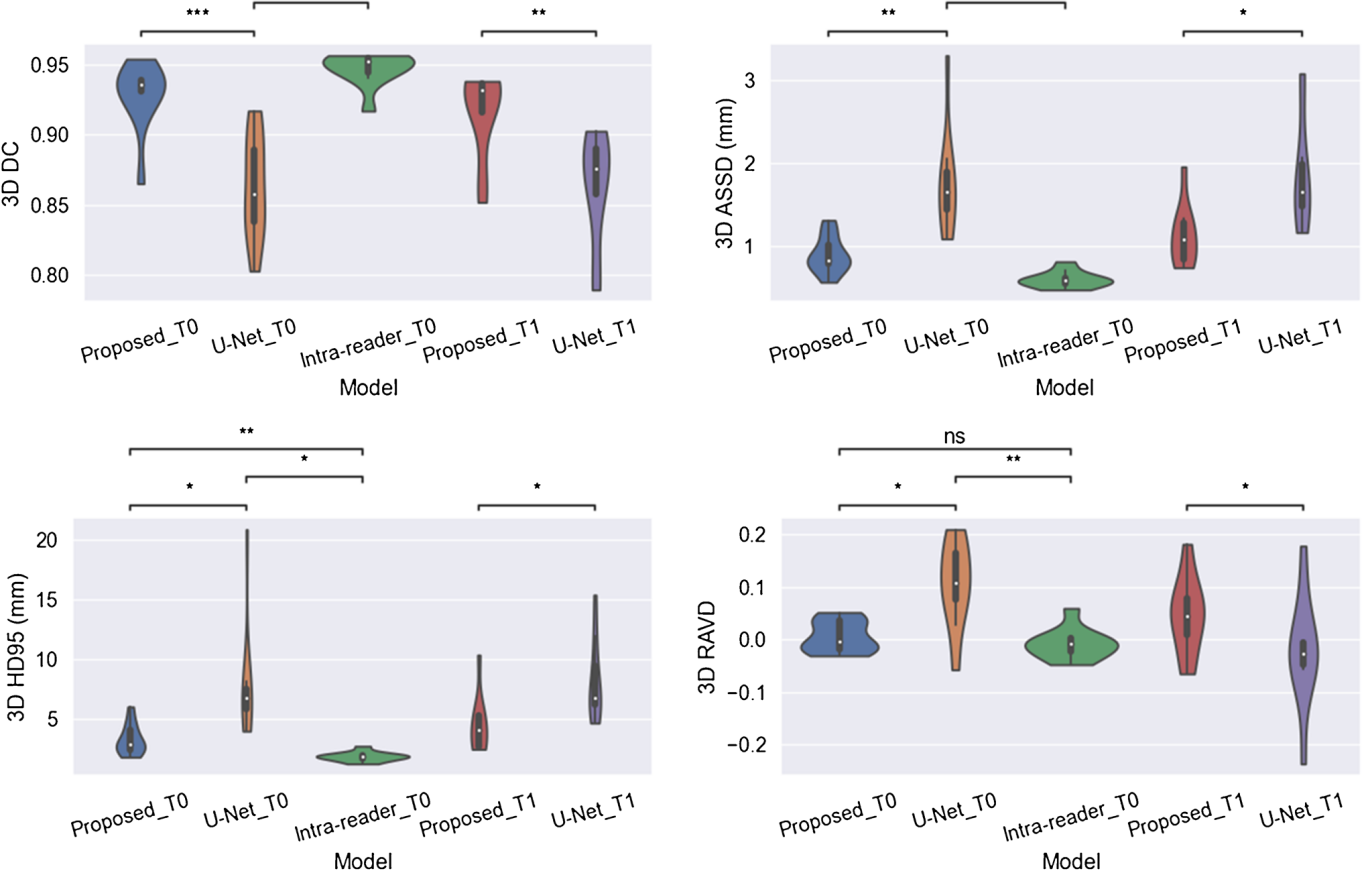
Violin plots for performance comparison of the three methods in Dice coefficient (DC), average symmetric surface distance (ASSD), 95th percentile Hausdorff distance (HD95), and relative average volume difference (RAVD), respectively. *p*-values are from paired *t*-tests. Significance levels: ns (not significant), *p < 0.05, **p < 0.01), ***p < 0.001, ****p < 0.0001

**Fig. 4:**
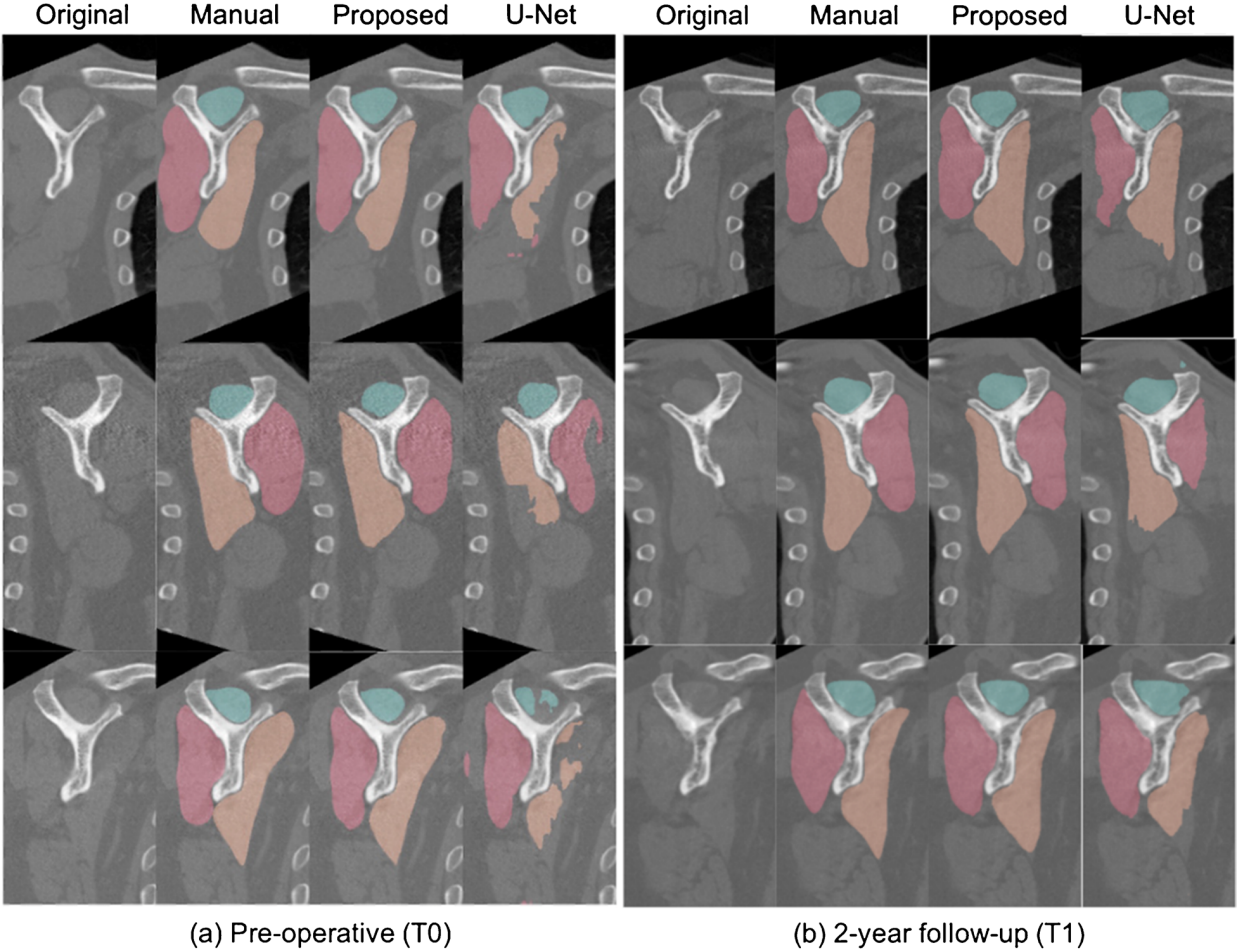
Examples of rotator cuff muscle segmentation on CT slices collected at **a** pre-operative and **b** 2-year follow-up from three test cases with different segmentation methods. Supraspinatus is depicted in green; infraspinatus/teres minor is in red; and subscapularis is in orange

The segmentation results are summarized in Table 1. Specifically, for the T0 CT scans, the proposed model achieved a mean Dice of 0.928, a mean ASSD of 0.844 mm, a mean HD95 of 3.071 mm, and a mean RAVD of 0.025 on the hold-out test set; in comparison, the benchmark 2D U-Net had a mean Dice of 0.863, a mean ASSD of 1.642 mm, a mean HD95 of 7.229 mm, and a mean RAVD of 0.119; and the intra-reader variation had a mean Dice of 0.947, a mean ASSD of 0.554 mm, a mean HD95 of 1.681 mm, and a mean RAVD of 0.022. Similarly, for the T1 CT scans, the proposed model achieved a mean Dice of 0.916, a mean ASSD of 1.028 mm, a mean HD95 of 4.173 mm, and a mean RAVD of 0.068 on the hold-out test set; in comparison, the benchmark U-Net had a mean Dice of 0.863, a mean ASSD of 1.882 mm, a mean HD95 of 8.084 mm, and a mean RAVD of 0.071. More detailed distribution of the evaluation metrics is depicted in violin plots in Fig. 3. Segmentation examples of the three rotator cuff muscle groups on sample slices from three test cases using the different segmentation methods at T0 and T1 time points are shown in Fig. 4a and b, respectively.

**Table 1:** Segmentation performance comparison among different methods on the hold-out test sets at pre-operative (T0) and minimum 2-year follow-up (T1) time points. Evaluation metrics used include Dice similarity score, average symmetric surface distance (ASSD) in millimeter, 95th percentile Hausdorff distance (HD95) in millimeter, and relative absolute volume difference (RAVD). *p*-values are from paired *t*-tests. Significance levels: ns (not significant), *p < 0.05, **p < 0.01, ***p < 0.001, ****p < 0.0001

	Dice	ASSD (mm)	HD95 (mm)	RAVD
Pre-operative (T0)				
U-Net	0.863	1.642	7.229	0.119
Proposed	0.928	0.844	3.071	0.025
Intra-reader	0.947	0.554	1.681	0.022
* p (proposed vs U-Net*	**** 5.6e − 04	** 2.56e − 03	* 4.13e − 02	* 2.11e − 02
* p (proposed vs intra-reader)*	* 1.13e − 02	** 2.58e − 03	** 9.19e − 03	ns 1.00e + 00
* p (U-Net vs intra-reader)*	**** 9.78e − 05	*** 8.97e − 04	* 1.42e − 02	** 1.44e − 03
Minimum 2-year follow-up (T1)				
U-Net	0.863	1.882	8.084	0.071
Proposed	0.916	1.028	4.173	0.068
* p (proposed vs U-Net)*	** 1.33e − 03	* 1.18e − 02	* 1.04e − 02	* 4.48e − 02

### Fat fraction quantification

**Fig. 5:**
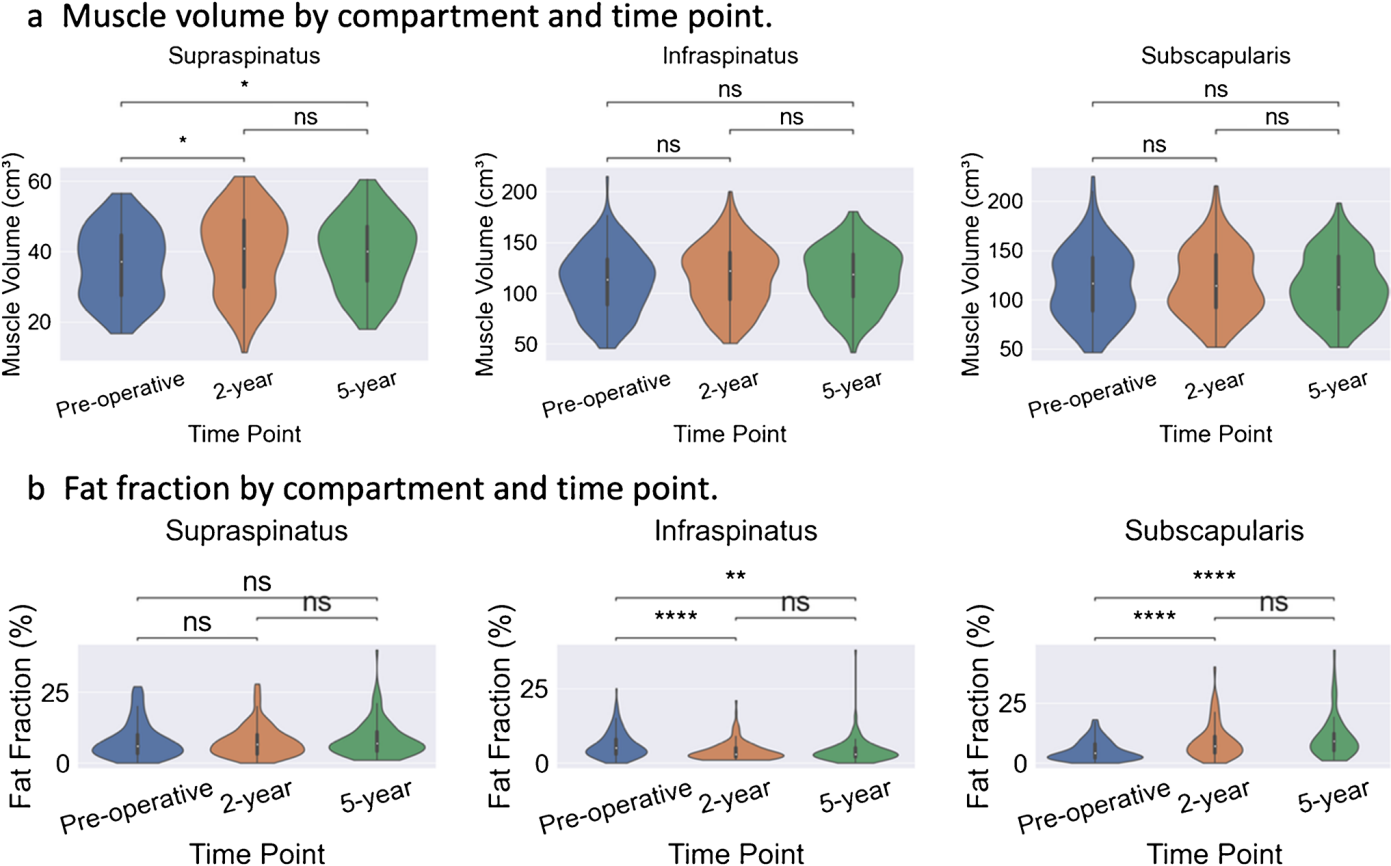
Violin plots of compartment-wise muscle volumes and fat fractions. **a** Comparison of muscle volumes (cm.^3^) at pre-op, 2-year, and 5-year time points for supraspinatus, infraspinatus/teres minor, and subscapularis, respectively. **b** Comparison of fat fractions (%) at pre-op, 2-year, and 5-year time points within supraspinatus, infraspinatus/teres minor, and subscapularis, respectively. *p*-values are from paired *t*-tests. Significance levels: ns (not significant), *p < 0.05, **p < 0.01, ***p < 0.001, ****p < 0.0001

**Fig. 6:**
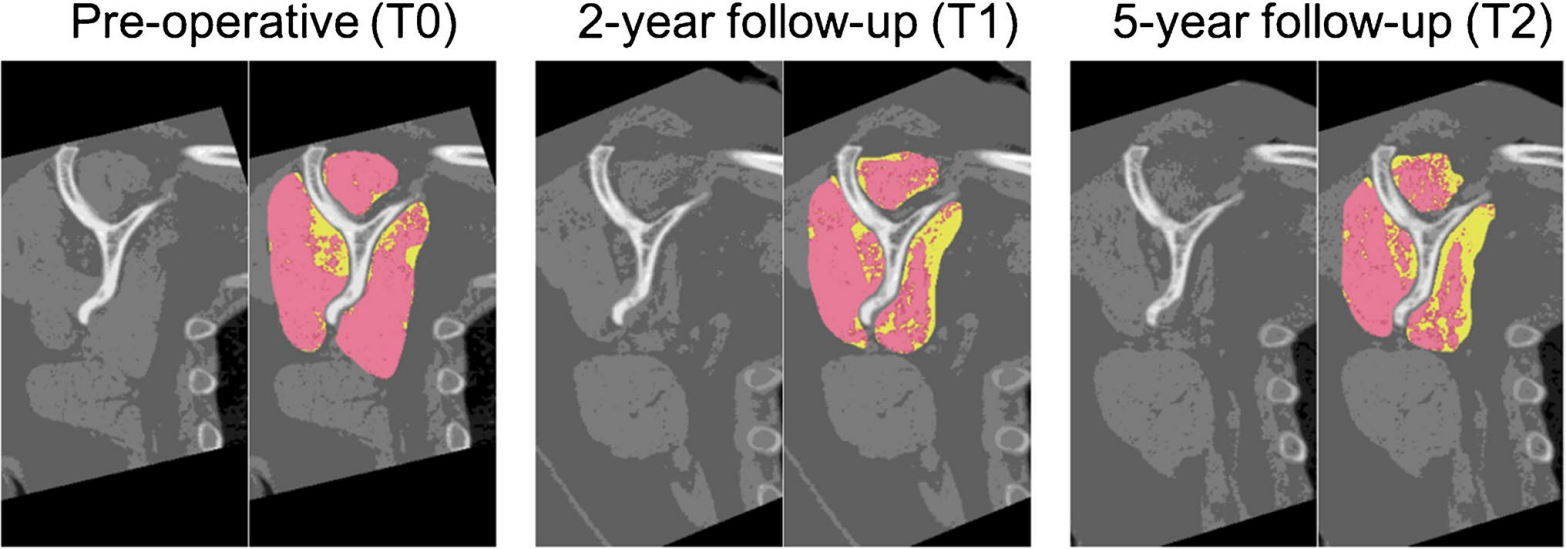
Sample slices from a random patient showing the muscle and fat segmentation of supraspinatus, infraspinatus/teres minor, and subscapularis at pre-op scan (T0), 2-year (T1), and 5-year follow-up (T2) scans, where muscle is in red and fat is in yellow

Violin plots showing compartment-wise muscle volumes and FF distributions after applying the trained models to the entire cohort for CT scans collected at three different time points are shown in Fig. 5a and b, respectively. Sample CT slices showing muscle and fat segmentations from a random patient at T0, T1, and T2 time points are depicted in Fig. 6, where muscle is in red and fat is in yellow.

## Discussion

Muscle volume and fat fraction are critical factors in the prognosis and treatment of shoulder disorders [23–25]. Despite their importance, automated methods for segmenting and evaluating rotator cuff muscle volumes from CT images remain underexplored. This study investigated the feasibility of developing automated segmentation models to quantify 3D rotator cuff muscle volumes and fat fractions in patients undergoing TSA. Specifically, the proposed muscle segmentation models in this study adopted DeepLabV3 + with ResNet50 as the backbone and achieved superior performance on both pre-operative and 2-year follow-up CT scans. The trained segmentation models were then successfully applied to preoperative and minimum 2- and 5-year follow-up CT scans from the entire 172 patient cohort. Three-dimensional muscle and fat volumes and fat fractions were computed and visualized.

As shown in Fig. 3, the segmentation performance of the proposed model for pre-operative CT scans outperformed the benchmark 2D U-Net model in all four evaluation metrics utilized in the study. The proposed model achieved the best performance in cases with clear muscle boundaries and minimal imaging artifacts, as illustrated in Fig. 4a row 1, where muscle volume segmentation closely matched the reference. In contrast, the 2D U-Net performed worst in cases affected by beam hardening artifacts Fig. 4a, row 2 and severe fatty infiltration Fig. 4a, row 3, leading to under-segmentation and boundary leakage. These failures are likely due to the 2D model’s limited contextual understanding and sensitivity to local intensity variations, which the proposed model mitigates through enhanced feature learning and robustness to intensity heterogeneity. The proposed model also performed favorably compared to intra-reader variation seen with manual segmentation. In particular, the RAVD, which is a clinically relevant metric looking at muscle volume differences, matched well between the proposed model and intra-reader variation. Following automated segmentation with the proposed models, the average muscle volume distribution remained similar across the three time points for each rotator cuff muscle group (supraspinatus, infraspinatus/teres minor, and subscapularis), as shown in Figs. 5 and 6. The fat fraction within the subscapularis muscle, however, showed an increasing trend with longer postoperative time Figs. 5 and 6. Statistical comparisons Table 2 revealed distinct longitudinal trends in rotator cuff muscle volume and fat fraction following total shoulder arthroplasty. Supraspinatus muscle volume showed significant increases from pre-operative to both 2-year and 5-year follow-ups (*p* < 0.01), suggesting potential recovery or compensatory hypertrophy in this muscle. In contrast, infraspinatus and subscapularis volumes did not exhibit significant changes across time points, which may indicate limited muscle remodeling or surgical impact in these compartments. Fat fraction analysis further highlighted differential patterns among muscles. The subscapularis exhibited significant increases in fat content from pre-operative to later follow-ups (*p* < 0.0001), consistent with progressive fatty infiltration and muscle degeneration—factors known to impair clinical outcomes. Supraspinatus fat fraction, however, remained statistically unchanged, possibly reflecting early intervention or less severe baseline degeneration. This increase in fatty infiltration of the rotator cuff is consistent with prior studies that have shown secondary rotator cuff dysfunction or failure can occur after anatomic TSA and may be a cause of eventual implant failure and need for revision surgery over time [46]. Reliable and automated methods to quantify muscle volume and fat fraction like the current technique can be used to better understand how these rotator cuff muscle changes develop in the short and long term after TSA and be used to impact TSA planning and treatment decision-making, including the type of shoulder arthroplasty (anatomic versus reverse TSA) selected for surgery and in developing better postoperative rehabilitation protocols and surgical repair techniques that optimize rotator cuff function postoperatively. These findings underscore the importance of compartment-specific analysis in assessing muscle health and post-surgical recovery. The observed differences may be influenced by variations in surgical exposure, loading patterns during rehabilitation, or intrinsic vulnerability to degeneration. Future work should explore the relationship between these changes and functional outcomes to better guide personalized patient management.

**Table 2:** Statistical comparison of rotator cuff muscle volume and fat fraction across time points. Pairwise *t*-tests were conducted between pre-operative, 2-year, and 5-year time points for each muscle (supraspinatus, infraspinatus, subscapularis). Significance is denoted as ns (not significant), *p < 0.05, **p < 0.01, ***p < 0.001, and ****p < 0.0001

	Supraspintus	Infraspinatus	Subscapularis
Muscle volume			
* p* (pre-op vs 2-year)	* 4.5e − 02	1.05e − 01	6.97e − 01
* p* (pre-op vs 5-year)	** 7.5e − 03	2.01e − 01	5.52e − 01
* p* (2-year vs 5-year)	8.67e − 01	7.77e − 01	8.34e − 01
Fat fraction			
* p* (pre-op vs 2-year)	7.31e − 01	**** 6.54e − 06	**** 1.05e − 08
* p* (pre-op vs 5-year)	7.37e − 01	** 4.04e − 03	**** 9.47e − 12
* p* (2-year vs 5-year)	4.91e − 01	7.47e − 01	7.61e − 02

Few studies have investigated automated rotator cuff muscle segmentation on clinical CT images. A recent paper reported a mean 2D Dice of 0.88 using U-Net on cross-sectional slices, defined as the plane perpendicular to the scapular axis passing through the spinoglenoid notch [26]. In comparison, our model achieved a mean 3D Dice of 0.928 on the entire muscle volume anatomically. Intuitively, DeepLabV3 + with ResNet50 as the backbone of our models offers advantages in feature extraction from ResNet50 and multi-scale spatial context handling with the Atrous convolutions, making it better suited for complex segmentation tasks. In addition, we further reported metrics like ASSD, HD95, and RAVD, which are complementary to Dice. It is worth noting that there have been multiple studies applying deep learning models to rotator cuff muscle segmentation tasks on MRIs [27–30]. In particular, Medina et al. reported a 2D Dice of 0.98 on the cross-sectional “Y-view” slice using a 2D U-Net. This exceptional performance is largely due to the superior soft tissue contrast of MRI, which provides anatomically rich data compared to CT, and the use of preprocessing techniques such as contrast-limited adaptive histogram equalization to enhance image quality. Riem et al. reported a 3D Dice of 0.92 for muscle volume segmentation on MRI using modified 3D U-Net models, achieving results comparable to ours in this study. However, these MRI-based approaches often focused primarily on cross-sectional segmentation of a single oblique sagittal slice (typically the Y-view) to estimate muscle area and fatty infiltration. In contrast, our CT-based pipeline enables full 3D volumetric quantification of muscle and fat using routinely acquired clinical shoulder CT scans. While CT is less sensitive to subtle soft tissue differences than MRI, it is more widely available in pre- and postoperative shoulder imaging, especially for total shoulder arthroplasty patients. Metal artifact reduction techniques also allow for postoperative CT imaging of patients following TSA, while more significant image distortion can be present with postoperative MRI following shoulder arthroplasty. Our method leverages this availability by using deep learning to extract high-resolution 3D muscle features despite lower inherent tissue contrast. This makes our approach more scalable and clinically feasible for longitudinal monitoring, particularly in surgical cohorts where MRI may not be routinely acquired. Moreover, volumetric CT-based measures may offer better sensitivity to detect regional variations in muscle atrophy and fatty infiltration than single-slice MRI metrics.

Note that we chose a 2D DeepLabV3 + architecture with a ResNet50 backbone over 3D models like 3D U-Net and nnU-Net based on two key considerations. First, the reference segmentations were provided as 2D annotations rather than full 3D volumes, making a 2D model a natural fit for the available ground truth. Second, although 3D architectures such as 3D U-Net or nnU-Net are commonly used for volumetric medical image segmentation, applying them to full-resolution shoulder CT volumes would require substantial downsampling or patching due to GPU memory limitations. This downsampling could compromise anatomical detail required for accurate muscle and fat boundary delineation. The 2D model allowed us to preserve full in-plane resolution and leverage pretrained backbones for efficient and high-fidelity slice-wise segmentation.

Despite promising results, several limitations remained in this study which will be a focus of future work. First, the current study lacked external validation. In future work, external validation datasets will be obtained to further evaluate the model performance. Second, the current study relied on manual selection of landmarks for reformatting the CT scans and selecting the starting and ending slices for muscle segmentation and volume calculation. Automated selection of these landmarks will be developed and built into this algorithm. Third, this study relied on manual segmentations performed by a single rater, and therefore, inter-reader variability was not assessed. While intra-reader testing indicated good consistency, the absence of multi-rater ground truth introduces potential bias and limits the ability to assess annotation robustness across observers. Future work incorporating multiple expert annotators will be important to quantify inter-reader agreement and further validate model performance. Despite the limited size of the training dataset, the deep learning model demonstrated high segmentation accuracy, likely due to the consistency of the annotations, standardized preprocessing, and the relatively constrained anatomical region of interest. Nevertheless, the modest dataset size remains a limitation and may impact the generalizability of the model. Future work using larger and more diverse datasets from multiple institutions will be necessary to further validate and improve model robustness. In addition, some CT scans were of low quality and/or contained artifacts, such as beam hardening. Although the segmentation model was able to handle these artifacts and return satisfactory segmentations, these scans had to be manually examined and excluded from the study for muscle volume and fat fraction quantification. Finally, this work focused on validating the automation of 3D muscle segmentation for rotator cuff muscle volume and fat fraction quantification. Further analysis of quantitative 3D muscle volume and fat fraction together with other clinical variables as predictors of patient-reported outcomes following TSA in this patient cohort will be undertaken. Future work developing an automated algorithm to identify and exclude low quality CT scans is planned, as well as transferring the models developed herein to quantify longitudinal changes in 3D muscle volume and fat fraction in RCR patients.

## Conclusion

This study developed accurate and reliable automated rotator cuff muscle segmentation models for clinical CT images in a TSA cohort based on deep learning. These models can substantially reduce the time required for quantification of 3D rotator cuff muscle volume and fat fraction analysis, allowing a deeper understanding of the relationship between muscle pathology and clinical outcomes following shoulder procedures such as TSA and RCR. Rotator cuff muscle atrophy and fatty infiltration have been correlated with worse clinical outcomes after RCR and TSA, but are still incompletely understood. Reliable methods to quantify muscle volume and fat fraction will provide physicians powerful tools to optimize patient management and improve patient outcomes in RCR and TSA, including developing better postoperative rehabilitation protocols and surgical repair techniques.

## Data Availability

The data used in this study cannot be shared publicly due to privacy and confidentiality associated with Protected Health Information (PHI). For further inquiries, please contact the corresponding author at yangm@ccf.org.
